# Work Status, Absenteeism, Presenteeism, and Quality of Life in Young Adult Cancer Survivors

**DOI:** 10.1001/jamanetworkopen.2025.28882

**Published:** 2025-08-26

**Authors:** Neel S. Bhatt, Jenna Voutsinas, Mackenzie Winters, Wendy M. Leisenring, Sheri Ballard, Kari Jenssen, K. Scott Baker

**Affiliations:** 1 Clinical Research Division, Fred Hutchinson Cancer Center, Seattle, Washington; 2 Department of Pediatrics, University of Washington, Seattle; 3 University of Washington School of Medicine, Seattle

## Abstract

**Importance:**

Current literature lacks information on the association between the work status and performance of young adult cancer survivors and their quality of life (QOL).

**Objective:**

To assess self-reported work status, missed time at work (absenteeism), and performance (presenteeism) and their associations with QOL among young adult cancer survivors.

**Design, Setting, and Participants:**

This cross-sectional survey study was performed from October 18, 2020, to September 17, 2022, at a single cancer center in Seattle, Washington. Participants included young adult cancer survivors (aged 18 to 39 years at diagnosis) who were 1 year or more from completion of cancer therapy. Data were analyzed from May 1, 2023, to February 1, 2025.

**Exposures:**

Cancer therapy.

**Main Outcomes and Measures:**

Unemployment rate compared with the age-, sex-, and calendar year–matched general population; standardized absolute and relative absenteeism and presenteeism scores; standardized scores for domains of anxiety, depression, physical function, fatigue, sleep disturbance, pain, social role, and cognition; and adjusted linear regressions to study factors associated with higher QOL scores.

**Results:**

A total of 198 survivors, with a median age at diagnosis of 31 (IQR, 26-35) years and a median age at survey of 39 (IQR, 35-44) years were included (142 [71.7%] female). The unemployment rate (14 [7.1%]) did not differ from that of the general population (4.7%) (*P* = .13). Compared with employed survivors, unemployed survivors reported significantly higher depression scores (coefficient, 5.11; 95% CI, 0.92-9.30) and lower scores for satisfaction with social roles and activities (coefficient, −6.59; 95% CI, −11.34 to −1.84) and physical function (coefficient, −6.63; 95% CI, −11.38 to −1.87). Higher absolute presenteeism score was associated with lower scores for anxiety (coefficient, −0.19; 95% CI, −0.26 to −0.11), depression (coefficient, −0.17; 95% CI, −0.24 to −0.10), fatigue (coefficient, −0.20; 95% CI, −0.30 to −0.10), pain interference (coefficient, −0.11; 95% CI, −0.21 to −0.02), and sleep disturbance (coefficient, −0.12; 95% CI, −0.21 to −0.03) and higher scores for physical function (coefficient, 0.08; 95% CI, 0.008-0.17), cognitive function (coefficient, 0.19; 95% CI, 0.11-0.27), and satisfaction in social roles and activities scores (coefficient, 0.16; 95% CI, 0.08-0.24). Additionally, a higher absolute absenteeism score was associated with a higher anxiety (coefficient, 0.03; 95% CI, 0.004-0.07) and lower cognitive function (coefficient, −0.04; 95% CI, −0.07 to −0.004).

**Conclusions and Relevance:**

In this cross-sectional study of young adult cancer survivors, associations were found between survivors’ self-reported work status and performance and their QOL affecting their mental, physical, and social health. These findings call for the development of effective communication strategies with employers to balance work expectations with survivors’ treatment-related complications to achieve better performance and in turn higher QOL.

## Key Points

**Question:**

Are there any associations between the work status and performance of young adult cancer survivors and their physical, mental, and social function?

**Findings:**

In this cross-sectional survey study including 198 young adult cancer survivors, approximately 1 in 10 reported being unemployed, and unemployment was associated with self-reported adverse physical, mental, and social health. Among those employed, better work performance was associated with better quality of life in all domains, while higher missed time at work was associated with adverse mental health.

**Meaning:**

These findings underscore the need for interventions to improve work status, performance, and quality of life of young adult cancer survivors.

## Teaser

This cross-sectional study assesses self-reported work status, work absenteeism, and work performance and their association with quality of life among young adult cancer survivors.

## Introduction

Nearly 70 000 young adults (aged 18-39 years at diagnosis) are diagnosed with cancer in the US every year.^1^ When compared with survival gains made in children and older adults with cancer over time, their survival outcomes have not shown similar improvement.^2^ Stagnant outcomes among young adults with cancer are thought to be related to several diverse factors, including differences in disease biology, concerns related to health care access, availability of caregivers, medication therapy adherence, and unequal access and participation in clinical trials.^3,4^ Young adults who survive cancer are also at a high risk of late psychosocial complications due to their disease presenting at a critical personal and professional development phase, particularly involving transitioning into adult roles, which could impact their educational and/or vocational attainment, relationships, financial independence, and peer support.^5^

Patient and clinician focus on completion of cancer-directed therapy should not neglect the unique challenges that survivors continue to face even after achieving disease remission, including their ability to integrate back into their routine lives.^6,7,8,9,10^ Specifically, returning to work after completion of therapy is an important milestone for previously employed survivors, serving as an indicator of overall social and economic well-being.^10^ Additionally, some younger adult survivors may have difficulties finding or maintaining employment once they are eligible, due to the disruption in education and progress toward getting employed likely due to preexisting and developing chronic health conditions.^11,12^ Last, survivors’ employment status may be impacted by external factors such as the COVID-19 pandemic^13^ and related economic impact and should be considered. Occupational and vocational pursuits not only serve an important role for survivors’ sense of identity, they also play a significant role in survivors’ financial stability and quality of life (QOL) after treatment. Specifically, unemployment could have a direct impact on survivor’s insurance status, as almost 50% of the US population depends on employment for their insurance coverage.^14^ Unemployment and resultant lack of insurance access can potentially lead to financial hardship, limited access to a healthy lifestyle and optimal screening and follow-up, and resultant adverse QOL.^4^ Previous research has shown that high unemployment rates and difficulties in returning to work are common among survivors of childhood cancer^6^ and older adults.^7^ However, evidence on work outcomes and QOL of young adult cancer survivors remains lacking. Moreover, it is possible that employed survivors often miss time at work due to scheduled clinic visits or unexpected illnesses, which may subsequently impact their overall income potential and need to be studied.

We conducted a cross-sectional survey study to assess self-reported work status, missed time at work (absenteeism), and work performance (presenteeism) and their association with QOL among young adult cancer survivors. We hypothesized that survivors’ unemployment would be directly associated with poor QOL. Additionally, we hypothesized that higher work absenteeism and lower presenteeism would be associated with poor QOL for those survivors working at the time of the survey. Additionally, the study aimed to assess the cost of lost work productivity from the survivors’ perspective in this population.

## Methods

### Study Population and Recruitment

Between October 18, 2020, and September 17, 2022, we conducted a cross-sectional web-based survey study of young adult cancer survivors treated at the Fred Hutchinson Cancer Center, Seattle, Washington. Patients aged 18 to 39 years at diagnosis of cancer and 1 or more years past completion of primary cancer-directed therapy were approached. Study participants had to be proficient in English to participate in the survey. The Institutional Review Board at Fred Hutchinson Cancer Center approved the study. This study followed the Strengthening the Reporting of Observational Studies in Epidemiology (STROBE) reporting guideline for cross-sectional studies. After obtaining Institutional Review Board approval, eligible survivors were approached by regular mail or email. They were provided with the link to the study website, from where they were able to access the Research Electronic Data Capture (REDCap) website. Interested participants provided online informed consent and accessed the web-based survey on the REDCap website. Follow-up attempts were made by either email and/or telephone calls as needed. All respondents were offered a $20 gift card as an incentive for their participation. Medical records of survivors with complete survey responses were abstracted for their sociodemographic, disease, and treatment details.

Race and ethnicity data were abstracted from the study participants’ medical records. Race was categorized as American Indian or Alaska Native, Asian, Black or African American, Native Hawaiian or Other Pacific Islander, White, or multiracial; ethnicity was categorized as Hispanic or non-Hispanic. These data were collected because of potential differences in employment and related outcomes due to race and ethnicity.

### Survey Domains and Outcomes

All participants completed an online version of the World Health Organization Health and Work Performance Questionnaire and the National Institutes of Health Patient Reported Outcomes Measurement Information System (PROMIS) computerized adaptive test surveys. The Health and Work Performance Questionnaire was used to assess survivors’ self-reported work status, type of work, absenteeism, presenteeism, and demographic characteristics such as age at the time of survey, marital status, caregiver status, number of children, highest educational attainment, and annual household income. Additional questions were used to determine the association between survivors’ absenteeism and their cancer history, such as absence at work due to cancer or cancer treatment. Those survivors who reported being students answered questions regarding missed time at school and school performance. Survivors who reported to be both students and workers only answered questions related to their work. Study participants completed the National Institutes of Health PROMIS computerized adaptive test measures for self-reported anxiety, depression, physical function, fatigue, sleep disturbance, pain, satisfactions with social role and activities, and cognitive function domains. Last, since the study was conducted at the height of the COVID-19 pandemic, an optional survey aiming to understand the role of COVID-19 among survivors, their family, and their work and school performance was also included.

### Scoring

The absolute absenteeism score was derived by subtracting the number of self-reported hours survivors worked at their workplace from the number of hours they were expected to be at work in the last 4 weeks. The scores ranged from a negative value (working more than expected) to a maximum positive upper bound of hours expected at work (always absent). The relative absenteeism score was derived by dividing the absolute absenteeism score by the number of hours expected at work in the last 4 weeks. The scores also ranged between a negative number (working more than expected) to 1.0 (always absent). Higher absenteeism scores indicated higher missed time at work. The absolute presenteeism score was derived by multiplying self-reported work performance by 10. It had a lower bound of 0 (total lack of performance) and an upper bound of 100 (maximum performance). The relative presenteeism score was derived by dividing survivors’ self-reported work performance by survivor-reported work performance of coworkers on the same scale. The scores ranged from 0.25 to 2.00, where 0.25 was the worst relative performance (≤25% of other workers’ performance) and 2.00 was the best performance (≥200% of other workers’ performance). Higher presenteeism scores indicated higher work performance.

PROMIS domains were scored on a T-score metric with a mean score of the reference population at 50 and SD of 10. For negatively worded domains (anxiety, depression, fatigue, sleep disturbance, and pain interference), a higher score was considered worse; for positively worded domains (cognitive function, physical function, and satisfaction with social roles and activities), a lower score was considered worse.

### Statistical Analysis

Data were analyzed from May 1, 2023, to February 1, 2025. Categorical variables were described using frequency and percentages and continuous variables using median and IQR. To compare our sample’s unemployment rate with that of the general population, we calculated the expected number of unemployed people in our cohort based on age, sex, and month and year of survey-specific unemployment rates from the US Bureau of Labor Statistics^15^ and compared the number of observed vs expected unemployed people in our population using the exact binomial test.

To calculate participants’ earnings lost because of missed time at work secondary to cancer or cancer treatment, we obtained the annual per capita income from the US Census website,^16^ matching each participant on zip code and race. We divided that value by 240 (average nonholiday and/or nonvacation days worked per year) to derive the mean income per day per person and multiplied the result by the number of days the participant was reported to have missed due to cancer or cancer treatment in the past year.

To study associations between demographic, disease-related, and employment-related variables and the PROMIS domains, we used linear regression models with the PROMIS scores as outcomes and each potential risk factor in separate models adjusted for age at survey, sex, and race and ethnicity, selected a priori based on literature review.^7,17^ Tests for interactions between sex and work-related variables were evaluated. To account for incomplete survey responses, we used multiple imputation by predictive mean matching to create multiple imputed datasets. For analyses involving work-related variables, patients with all missing values for employment questions were not imputed, nor were patients who indicated they were unemployed or students. Regression results were pooled using Rubin’s rules to obtain pooled estimates, 95% CIs, and *P* values.^18^ Two-sided *P* < .05 was considered statistically significant. We also used the same methods to look at absolute and relative presenteeism and absenteeism and unemployment as outcomes. More detailed information on the imputation process can be found in the eAppendix in Supplement 1. All analyses were completed using R, version 4.1.3 (R Program for Statistical Computing).

## Results

### Respondent Characteristics

Among 1067 survivors who were approached for the survey, 375 initiated the survey (35.1% response rate). After excluding ineligible participants (n = 43) and incomplete responses (n = 134), a total of 198 of 332 respondents (59.6% survey completion rate) were included in the analysis (eFigure in Supplement 1). Survey respondents’ median age at diagnosis and survey was 31 (IQR, 26-35) years; at survey, 39 (IQR, 35-44) years. One hundred forty-two respondents (71.7%) were female and 56 (28.3%) were male. The most common clinical diagnoses were hematologic cancer (53 of 194 [27.3%]) and breast cancer (53 of 194 [27.3%]). Most survivors reported having annual household income of greater than $100 000 (112 of 190 [58.9%]) and being married or living with a partner (133 of 194 [68.6%]) (Table 1).

**Table 1.** Baseline Characteristics of Survey Respondents

Characteristic	No. (%) of patients (N = 198)
Age at diagnosis, median (IQR), y	31 (26-35)
Age at survival, median (IQR), y	39 (35-44)
Sex	
Female	142 (71.7)
Male	56 (28.3)
Race	
American Indian or Alaska Native	9 (4.9)
Asian	51 (28.0)
Black or African American	6 (3.3)
Native Hawaiian or Other Pacific Islander	7 (3.8)
White	106 (58.2)
Multiracial	3 (1.6)
No. missing	16
Ethnicity	
Hispanic	26 (14.9)
Non-Hispanic	148 (85.1)
No. missing	24
Disease diagnosis	
Hematologic cancer	53 (27.3)
Breast cancer	53 (27.3)
Genital tract tumors	33 (17.0)
Sarcoma	7 (3.6)
Central nervous system tumors	6 (3.1)
Other	42 (21.6)
No. missing	4
Marital status	
Married or living with a partner	133 (68.6)
Other	61 (31.4)
No. missing	4
Highest educational level attained	
Bachelor’s degree or less	102 (56.0)
Master’s degree or higher	80 (44.0)
Missing	16
Most recent employment or educational status	
Employed	154 (77.8)
Student	3 (1.5)
Employed and student	9 (4.5)
Unemployed and looking for work	14 (7.1)
Other	18 (9.1)
Current occupation	
Professional (eg, engineer, accountant, systems analyst)	88 (56.1)
Precision production and crafts worker (eg, mechanic, carpenter, machinist)	2 (1.3)
Operator or laborer (eg, assembly line worker, truck driver, construction worker)	3 (1.9)
Sales (eg, sales representative, stockbroker, retail sales)	5 (3.2)
Service occupation (eg, security officer, food service worker, janitor)	8 (5.1)
Technical support (eg, laboratory technician, legal assistant, computer programmer)	9 (5.7)
Executive, administrator, or senior manager (eg, chief executive officer, sales vice president, plant manager)	26 (16.6)
Clerical and administrative support (eg, secretary, billing clerk, office supervisor)	16 (10.2)
No. missing	41
Treatment	
Surgery	
Yes	142 (72.4)
No	54 (27.6)
No. missing	2
Chemotherapy	
Yes	135 (68.5)
No	62 (31.5)
No. missing	1
Radiation therapy	95 (48.5)
No radiation	101 (51.5)
No. missing	2
Hematopoietic stem cell transplant	
Yes	22 (11.2)
No	175 (88.8)
No. missing	1
Annual household income, US $	
>100 000.00	112 (58.9)
60 000-99 999	39 (20.5)
<60 000	39 (20.5)
No. missing	8

In terms of race, 9 respondents (4.9%) were American Indian or Alaska Native, 51 (28.0%) were Asian, 6 (3.3%) were Black or African American, 7 (3.8%) were Native Hawaiian or Other Pacific Islander, 106 (58.2%) were White, and 3 (1.6%) were multiracial. In terms of ethnicity, 26 respondents (14.9%) were Hispanic and 148 (85.1%) were non-Hispanic.

### Unemployed Young Adult Cancer Survivors

Fourteen survivors (7.1%) reported being unemployed and looking for work. This rate was not significantly different from the unemployment rate in the general population of 4.7% (*P* = .13). Consistent with the entire cohort, most unemployed survivors were female (10 of 14 [71.4%]). However, only a small percentage of unemployed survivors reported completing master’s degrees or higher-level education (3 of 14 [21.4%]) or having an annual household income of greater than $100 000 (3 of 14 [21.4%]), which was different when comparing these frequencies with those of the entire cohort. In the adjusted univariate analysis, no associations between sociodemographic and treatment-related factors and unemployment were noted. Some of the survivors shared having difficulties finding employment due to a variety of factors, including physical and mental health challenges and the COVID-19 pandemic (eTable in Supplement 1).

### Work Performance Among Working Survivors

Of those survivors who were working, the median absolute absenteeism scores were 0.0 (IQR, −1.0 to 18.5) and relative absenteeism scores were 0.0 (IQR, 0.0-0.1), corresponding to no missed time at work. The median absolute presenteeism score was 80.0 (IQR, 69.2-88.0). The median relative presenteeism score was 1.0 (IQR, 0.9-1.2), corresponding to 100% work performance compared with their coworkers (Table 2). For respondents reporting 1 or more days of missed work due to their cancer or cancer treatment (51 of 157 [32.5%]), the calculated lost earnings were a median of $1262.7 (IQR, $627.0-$2758.3) per year, with the cumulative annual loss of earnings due to missed time at work for these respondents estimated at $152 209.

**Table 2.** Work Productivity Scores

Score category	Median score (IQR)	No. missing
Work productivity derived from WHO HPQ (n = 164)^a^		
Absolute absenteeism	0 (−1.0 to 18.5)	16
Relative absenteeism	0 (0.0 to 0.1)	16
Absolute presenteeism	80.0 (69.2 to 88.0)	22
Relative presenteeism	1.0 (0.9 to 1.2)	27
QOL Domain T (mean [SD], 50 [10]) derived from NIH PROMIS (n = 198)^b^		
Fatigue	55.5 (48.5 to 64.0)	0
Anxiety	58.0 (51.4 to 63.3)	0
Depression	52.2 (46.5 to 57.6)	0
Cognitive function	44.5 (39.0 to 50.4)	0
Physical function	52.2 (44.4 to 58.7)	0
Sleep disturbances	54.3 (46.5 to 59.5)	0
Pain interference	50.1 (38.7 to 56.0)	1
Satisfaction in social roles and activities	47.4 (44.0 to 54.1)	2

Abbreviations: HPQ, Health and Work Performance Questionnaire; NIH, National Institutes of Health; QOL, quality of life; PROMIS, Patient Reported Outcomes Measurement Information System; WHO, World Health Organization.

^a^ Higher absenteeism scores indicate higher missed time at work or school; higher presenteeism score indicates higher work productivity.

^b^ For negatively worded domains (anxiety, depression, fatigue, sleep disturbance, and pain interference), a higher score is considered worse; for positively worded domains (cognitive function, physical function, and satisfaction with social roles and activities), a lower score is considered worse.

Most survivors (n = 127 [85.2%; 95% CI, 78.5%-90.5%]) reported that their work performance was better than most coworkers in their job at some, most, or all of the time. However, 43 of the employed survivors (28.9% [95% CI, 21.7%-36.8%]) reported doing no work when work was expected some, most, or all of the time. Additionally, 44 of employed survivors (29.5%; 95% CI, 22.3%-37.5%) reported not working as carefully as intended some, most, or all the time. More than one-third (n = 53 [35.6%; 95% CI, 27.9%-43.8%]) believed that they had difficulty concentrating at work some, most, or all of the time. Half of the workers (n = 75 [50.0%; 95% CI, 41.7%-58.3%]) reported having no limitations at work due to health problems. When asked about the overall job performance in the last 4 weeks, more than two-thirds (n = 110 [73.3%; 95% CI, 65.5%-80.2%]) reported that their performance was a little to a lot better than that of their coworkers (Figure). Several survivors described challenges due to physical health, mental health, and external factors that adversely impacted their work-related challenges (eTable in Supplement 1). In regression analyses assessing self-reported work-related outcomes, we did not find any associations with any evaluated sociodemographic variables and therefore did not include results in Table 3.

**Figure.**
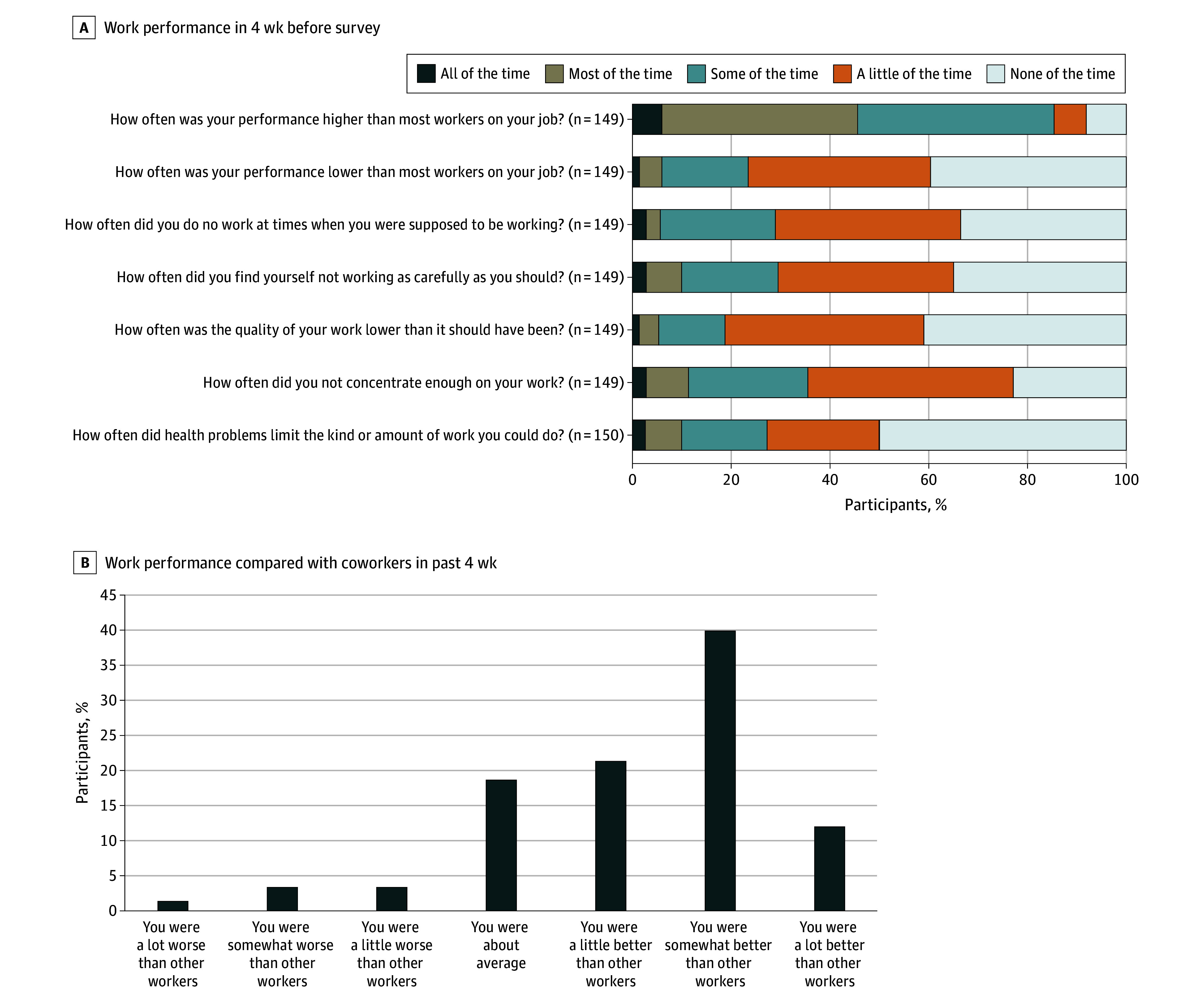
Self-Reported Work Performance Data are representative of 150 survey respondents.

**Table 3.** Pooled Linear Regression Results to Assess Factors Associated With PROMIS Domains^a^

PROMIS domain, variable^b^	Coefficient (95% CI)	*P* value
**Anxiety**		
Sex		
Female	[Reference]	NA
Male	−4.39 (−6.83 to −1.95)	<.001
Race		
Racial minority group	−2.94 (−5.27 to −0.61)	.01
White	[Reference]	NA
Absolute absenteeism	0.03 (0.004 to 0.07)	.03
Absolute presenteeism	−0.19 (−0.26 to −0.11)	<.001
**Cognitive function**		
Sex		
Female	[Reference]	NA
Male	4.52 (1.85 to 7.19)	.001
Chemotherapy		
No	[Reference]	NA
Yes	2.71 (0.18 to 5.24)	.04
Absolute absenteeism	−0.04 (−0.07 to −0.004)	.03
Relative absenteeism	−5.22 (−10.31 to −0.13)	.04
Absolute presenteeism	0.19 (0.11 to 0.27)	<.001
**Depression**		
Marital status		
Married or living with a partner	[Reference]	NA
Other	3.58 (1.23 to 5.93)	.003
Employment		
Employed	[Reference]	NA
Unemployed	5.11 (0.92 to 9.30)	.02
Absolute presenteeism	−0.17 (−0.24 to −0.10)	<.001
**Fatigue**		
Sex		
Female	[Reference]	NA
Male	−5.55 (−8.81 to −2.30)	.001
Marital status		
Married or living with a partner	[Reference]	NA
Other	3.26 (0.13 to 6.39)	.04
Educational level		
Bachelor’s degree or less	[Reference]	NA
Master’s degree or higher	−3.00 (−5.94 to −0.07)	.045
Absolute presenteeism	−0.20 (−0.30 to −0.10)	<.001
**Pain interference**		
Educational level		
Bachelor’s degree or less	[Reference]	NA
Master’s degree or higher	−4.31 (−7.28 to −1.34)	.005
Absolute presenteeism	−0.11 (−0.21 to −0.02)	.02
**Physical function**		
Sex		
Female	[Reference]	NA
Male	3.33 (0.52 to 6.14)	.02
Educational level		
Bachelor’s degree or less	[Reference]	NA
Master’s degree or higher	3.63 (1.13 to 6.13)	.005
Hematopoietic cell transplant		
No	[Reference]	NA
Yes	−6.21 (−10.09 to −2.34)	.002
Employment		
Employed	[Reference]	NA
Unemployed	−6.63 (−11.38 to −1.87)	.007
Absolute presenteeism	0.08 (0.008 to 0.17)	.053
**Satisfaction in roles and activities**		
Marital status		
Married or living with a partner	[Reference]	NA
Other	−4.00 (−6.67 to −1.34)	.003
Employment		
Employed	[Reference]	NA
Unemployed	−6.59 (−11.34 to −1.84)	.007
Absolute presenteeism	0.16 (0.08 to 0.24)	<.001
**Sleep disturbance**		
Marital status		
Married or living with a partner	[Reference]	NA
Other	4.25 (1.30 to 7.20)	.005
Absolute presenteeism	−0.12 (−0.21 to −0.03)	.01

Abbreviations: NA, not applicable; PROMIS, Patient Reported Outcomes Measurement Information System.

^a^ Results are from 5 imputed datasets. Each domain is adjusted for age at time of the survey, sex, and race and ethnicity. Variables evaluated in the models include age at diagnosis, age at time of the survey, sex, race and ethnicity, marital status, educational status, disease diagnosis, history of chemotherapy exposure, history of radiation therapy exposure, history of surgery, history of hematopoietic cell transplant, unemployment, absolute absenteeism, relative absenteeism, absolute presenteeism, and relative presenteeism.

^b^ Each row represents a single model. Only significant results are shown (*P* < .05).

### Factors Associated With Self-Reported QOL

Median T scores of all PROMIS domains are listed in Table 2. In the adjusted univariate regression analyses assessing the factors associated with each PROMIS domain, we noted that compared with employed survivors, unemployed survivors reported significantly higher depression scores (coefficient, 5.11; 95% CI, 0.92-9.30), lower satisfaction with social roles and activities (coefficient, −6.59; 95% CI, −11.34 to −1.84), and lower physical function scores (coefficient, −6.63; 95% CI, −11.38 to −1.87). We also identified an interaction between sex and unemployment on the depression score (interaction *P* = .04) such that unemployment was associated with higher scores for male patients than female patients. Higher absolute presenteeism scores were associated with lower scores for anxiety (coefficient, −0.19; 95% CI, −0.26 to −0.11), depression (coefficient, −0.17; 95% CI, −0.24 to −0.10), fatigue score (coefficient, −0.20; 95% CI, −0.30 to −0.10), pain interference (coefficient, −0.11; 95% CI, −0.21 to −0.02), and sleep disturbance (coefficient, −0.12; 95% CI, −0.21 to −0.03) as well as higher scores for physical function (coefficient, 0.08; 95% CI, 0.008-0.17), cognitive function (coefficient, 0.19; 95% CI, 0.11-0.27), and satisfaction in social roles and activities (coefficient, 0.16; 95% CI, 0.08-0.24). Additionally, a higher absolute absenteeism score was associated with a higher anxiety score (coefficient, 0.03; 95% CI, 0.004-0.07) and a lower cognitive function score (coefficient, −0.04; 95% CI, −0.07 to −0.004). Other factors associated with lower anxiety scores included male sex and racial minority group. Male survivors also reported higher scores for cognitive function and physical function and lower fatigue scores. Marital status other than being married or living with a partner was associated with higher scores for sleep disturbance, fatigue, and depression and lower scores for satisfaction in social roles and activities. Additionally, compared with the patients with attained education of a bachelor’s degree or less, those with a master’s degree or higher educational level reported lower scores for pain interference and fatigue and higher physical function scores. Last, a receipt of hematopoietic cell transplantation was associated with lower physical function. Nonsignificant results were not shown, but all variables tested are described (Table 3).

### COVID-19–Specific Data

A total of 159 survivors (80.3%, 95% CI, 74.1%-85.6%) completed the COVID-19–specific survey (Table 4). Sixty-five survivors (40.9%; 95% CI, 33.2%-48.9%) stated having very or extremely high anxiety regarding the COVID-19 pandemic. When assessing life events as a result of COVID-19, 43 survivors (27.0%; 95% CI, 20.3%-34.7%) reported experiencing a loss of job or reduced hours, 66 (41.5%; 95% CI, 33.8%-49.6%) reported experiencing missed or reduced number of visits with their health care team, and 45 (28.3%; 95% CI, 21.5%-36.0%) reported experiencing changes to their health care routine due to COVID-19.

**Table 4.** COVID-19–Related Responses From the Study Population

Variable	No. (%) of patients
Participation in COVID-19–specific survey	159 (80.3)
Has had symptoms of COVID-19 (all that apply)	
Patient	40 (25.2)
Family members	67 (42.1)
Other important persons	46 (28.9)
Has had a diagnosis of COVID-19 (all that apply)	
Patient	20 (12.6)
Family members	59 (37.1)
Other important persons	51 (32.1)
Treatment for COVID-19 (all that apply)	
Myself	5 (3.1)
Family members	28 (17.6)
Other important persons	30 (18.9)
Passed away from COVID-19 (all that apply)	
Family members	8 (5.0)
Other important persons	20 (12.6)
Anxiety around COVID-19 pandemic	
Not at all	9 (5.7)
Slightly	24 (15.1)
Moderate	61 (38.4)
Very	43 (27.0)
Extremely	22 (13.8)
Life events as a result of COVID-19 (all that apply)	
Loss of job or reduced hours	43 (27.0)
Missed or reduced visits with health care team	66 (41.5)
Changes to health care routine	45 (28.3)
Financial difficulties in covering basic needs	18 (11.3)
Other	17 (10.7)
How have changes in your life caused by COVID-19 impacted you?	
Changes have been all bad	5 (3.1)
Changes have been mostly bad	38 (23.9)
Some changes have been bad; some have been good	98 (61.6)
Changes have been mostly good	16 (10.1)

## Discussion

In this cross-sectional study of young adult cancer survivors, we found associations of self-reported work status and performance and QOL with physical, mental, and social health. Approximately 1 in 10 survivors was unemployed, and unemployment was associated with poor QOL. Moreover, among those who were employed, higher presenteeism was associated with better QOL, while higher absenteeism was associated with poor QOL.

The findings of our study have several implications. Given the potential impact of suboptimal work performance on the financial status of young adult survivors and its association with poor QOL, these findings highlight the need for routine longitudinal monitoring of work status, work performance, and QOL in this population by their health care team. Additionally, our study calls for multidisciplinary interventions to provide better psychosocial support for young adult cancer survivors as they recover from their treatment-related sequalae.

We did not identify significant differences in unemployment rates between young adult survivors and the general population, which is corroborated by prior studies assessing return to work after completion of therapy in young adult survivors in the US.^9,19^ Parsons et al^9^ used the Surveillance, Epidemiology, and End Results cancer registries and noted that approximately 72% of patients working or studying full-time prior to diagnosis had returned to work or school by 15 to 35 months after diagnosis. Another analysis from the Livestrong Survivorship Centers of Excellence Network reported that nearly 85% of the survivors were employed.^19^ In contrast, studies from Europe have shown higher rates of unemployment and disability compared with controls.^20,21,22^ Since most of the US population relies on employment for health insurance coverage,^14^ it is possible that survivors treated in the US may return to work after completion of therapy to maintain their insurance coverage and health care access.

Very few studies have assessed work productivity in young adult cancer survivors.^11,23^ Crespi et al^23^ found that more than one-quarter of the studied breast cancer survivors reported health-related work productivity loss. In contrast, Vetsch and colleagues^11^ reported that more than half of the survivors did not report compromised work performance. However, more than two-thirds noted impairment in vocational functioning compared with before the diagnosis. While our analysis lacked information on prediagnosis work performance, most survivors reported their work performance to be better than that of their coworkers. A small subset reported their work being of lower quality compared with coworkers, being less careful at work, and having some degree of limitation. It is possible that some survivors return to work or school too soon after completion of therapy, which may be overwhelming and lead to adverse impacts on their performance.^24^

We noted associations between both self-reported work status and performance and QOL. This was also reported in an analysis of working-age cancer survivors,^25^ which noted unemployment to be associated with higher pain, fatigue, sexual problems, social avoidance, appearance concerns, financial problems, cognitive problems, and negative feelings. In another study of adult survivors of childhood cancer,^26^ authors reported associations between unemployment and impaired task efficiency, somatization, and depression. Neither of these studies assessed the association between work performance and QOL in employed cancer survivors. Returning to work after completion of therapy is an important milestone, providing young adult survivors with structure, identity, and an independent role within society that has positive impacts on QOL.^10^ However, our study noted that despite most survivors being employed at the time of the study, several reported poor work performance and had associations with lower QOL, which requires attention.

One potential area of intervention could be optimizing psychosocial support during survivors’ return to work process after completion of therapy, since several challenges could emerge at that time. In fact, a few groups have focused their efforts on improving the return to work process for cancer survivors through various modalities such as educational materials, referral to relevant services, vocational counseling, promotion of physical activity, and social support, albeit with variable feasibility, acceptability, and efficacy.^27^ Additional work is needed to develop and test interventions to improve the understanding of young adult cancer survivors about their workplace rights through the use of self-efficacy tools such as Self-efficacy to Ask for Work Accommodations.^28^ Another area of opportunity for an intervention is in facilitating communication with employers to ensure adjustments in workplace expectations.^29^ This is especially important considering that prior research focused on breast cancer survivors has shown that accommodations provided by employers are associated with higher job retention in cancer survivors.^30^

### Limitations

Our analysis has several limitations that need to be acknowledged. It is known that young adult cancer survivors are typically underrepresented in research.^4^ While we assembled a large cohort of young adult cancer survivors in our analysis, it was a single-center analysis, and we were unable to conduct a multivariable analysis due to the limited number of events, such as number of unemployed survivors. While we speculate that the small number of unemployed survivors is likely due to the need for maintaining employer-sponsored insurance coverage, it is also possible that they were underrepresented in the analysis due to the need for internet and email access. Additionally, as this was a cross-sectional analysis and included survivors at various time points after completion of therapy, we may not be capturing overall risk of unemployment in this population. Our study used web-based patient-reported outcomes measures, and strengths and limitations of this approach have to be considered when interpreting the findings.^31^ Notwithstanding these limitations, our analysis still provides valuable information for clinicians on young adult cancer survivors’ unemployment and associated QOL-related challenges. Some of the survey responses were incomplete, and multiple imputation methodology was used to address that. When studying the cost associated with lost productivity, since the US Census data were limited to per capita income by zip code and race, and that the survey data gave income ranges and for household income only, it limited our ability to perform detailed analyses on participant income. We could not understand specific etiology behind the poor work performance and QOL due to lack of comprehensive chronic health conditions data. Given the cross-sectional design, the temporality of the study outcomes could not be understood. It is possible that the cost associated with lost productivity could lead to a higher financial hardship and resultant poor QOL.^32^ More research is needed to better understand the causes of poor work performance and QOL. Moreover, the impact of COVID-19 on cancer survivors’ mental health, finances, and overall health care access is known.^13,33,34^ It would be important to study these outcomes in the next few years to assess the postpandemic changes.

## Conclusions

In this cross-sectional study of young adult cancer survivors, unemployment was associated with higher risk of depression and lower satisfaction with social roles and activities, and higher missed time at work was associated with higher anxiety and lower cognitive function. Conversely, better self-perceived work performance was associated with better quality of life— specifically, lower risks of anxiety, depression, fatigue, pain interference, and sleep disturbance and better physical function, cognitive function, and satisfaction in social roles and activities. Based on these results, additional work must be done to further understand the work performance and QOL concerns by conducting qualitative in-depth interviews of young adult cancer survivors. Our findings also call for efforts to have routine assessment of survivors’ work-related challenges, financial hardship, and QOL, including physical, mental, and social health by health care professionals. Such studies will help set the stage for developing future strategies to prevent work-related complications and achieve better QOL for this population.

## Notes

Supplement 1. **eFigure.** CONSORT Diagram **eTable.** Patient Responses Regarding the Factors Associated With Work Status and Productivity **eAppendix.** Imputation

Supplement 2.
Data Sharing Statement
