# Supplementary material for: Work Status, Absenteeism, Presenteeism, and Quality of Life in Young Adult Cancer Survivors
Source: JAMA Netw Open. 2025 Aug 26;8(8):e2528882. doi: 10.1001/jamanetworkopen.2025.28882 (PMC12381670; doi:10.1001/jamanetworkopen.2025.28882)
Supplement: Supplement 1. — eFigure. CONSORT Diagram eTable. Patient Responses Regarding the Factors Associated With Work Status and Productivity eAppendix. Imputation [file jamanetwopen-e2528882-s001.pdf]

Supplementary Online Content

Bhatt NS, Voutsinas J, Winters M, et al. Work Status, Absenteeism, presenteeism, and quality of life in young adult cancer survivors. *JAMA Netw Open.* 2025;8(8):e2528882. doi:10.1001/jamanetworkopen.2025.28882

eFigure. CONSORT Diagram
eTable. Patient Responses Regarding the Factors Associated With Work Status and Productivity
eAppendix. Imputation

This supplementary material has been provided by the authors to give readers additional information about their work.

© 2025 Bhatt NS et al. *JAMA Network Open*.

eFigure. CONSORT Diagram

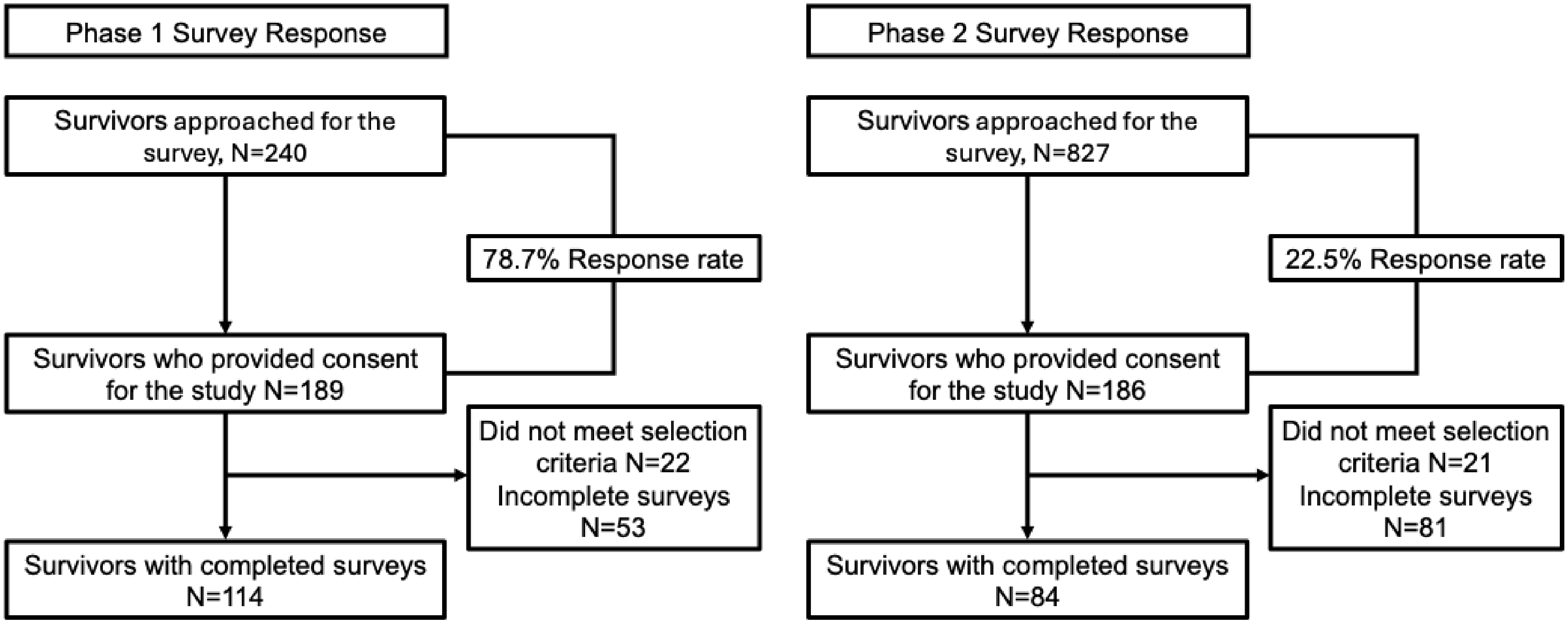

Phase 1 population: YA cancer survivors followed at our institution who had previously provided consent to be contacted for future research

Phase 2 population: all YA cancer survivors who were previously treated at our institution

© 2025 Bhatt NS et al. *JAMA Network Open*.

eTable. Patient Responses Regarding the Factors Associated With Work Status and Productivity

Factors	Selected responses from patients
**Physical health**	*"Specifically, marginal concerns that my cancer treatment had the potential long-term side effects of reduced lung function and a weaker immune system."*
	"It's also been so long since I've had cancer that I've adjusted to my energy levels. I'm guessing I'm more fatigued than I would have been had I not had cancer, but this is my normal now."
	"I've had near constant migraines since treatment, and they pretty much run my life. But I've done a lot too. My life isn't miserable, I just don't seem to have the same number of usable hours as everyone else."
	"My life has not been the same since my radiation treatment in 2019 my body hurts the simplest tasks cost me so much effort. My memory is so horrible I do not know what to do and it scares me having conversations in which I speak of a topic I had already spoken about."
	"A lot of my answers were influenced by the fact that we are currently living in a pandemic, and I am immunocompromised with other comorbidities. It affects what I can go out and do, and what social activity is possible. I am unemployed and completely dependent on my parents financially. I especially don't know how to find work during the pandemic because of my comorbidities, most of which relate to my cancer treatment. The focus on fatigue was very relevant to me."
	"I was very excited about my job as a bookseller at a local bookstore in 2020. However, when the pandemic hit, I realized I was in the category of at-risk people, due to my not having a spleen anymore (from surgery). That negatively impacted my job because I decided to stay home early on (even before most people began doing so). I currently do not work there anymore because I was let go. I am not as worried about catching the virus as I was in spring of 2020, but I do still feel concerned about the possibility of getting sick and becoming more ill than the average person, so I am very careful about my social interactions."
**Mental health**	*"No doubt the treatments and BMT prolonged my life but are now coming back to haunt me yet myself and my wife continue to remain resilient. We're 30 years post-transplant and my medical record continues to grow every year."*
	"I'd say it took me more (3 years) to recover mentally from the fact that I had cancer. It did change my perspective in life. In terms of career - progress came to stall due to worrying/anxiety all the time."
	"I think cancer has affected me mostly from my outlook on life and the anxiety that something bad is about to happen again."
	"This all affects my mental health right now especially in terms of anxiety. I also get fatigued easily since cancer which is even more frustrating being young and trying to work a 35-hour week in Education."
	"As a cancer survivor, I'm scared to death to go anywhere or be around people. People have been extremely aggressive and ruthless. The worst seems to have been brought out in people. I was already wallowing in a mounting depression when covid hit. Now I feel like everyone is down on my level."
**External factors**	*"I don't have any cancer-related pain, but I do have severe arthritis in my L knee (played college/pro basketball until I was 26, leading to wear and tear). As a result, I don't run anymore (but swim 3 miles a week, and often ride my bike)."*

© 2025 Bhatt NS et al. *JAMA Network Open*.

	"My answers maybe a little skewed due to just having a baby 3 months ago, regarding sleep. Also the fact I am now homeschooling my first grader due to the pandemic. I was okay before this."
	"My responses are influenced, not only by my cancer treatment and recovery, but also by the responsibilities I have caring for my wife, and two sons, the oldest of which is autistic. In short, I am always tired and can never get enough sleep, and as soon as I feel like I have found a sustainable balance, something changes. For example, in a few weeks, my oldest son will be on summer vacation, and I will undergo a minor knee surgery so the routines I have established now will not be applicable then and a new balance will have to be found."
	"Taking chemotherapy and radiation treatment - and how it actually cuts your budget by at least 35% due to recovery and fatigue and limited movement after the burns on your skin start happening from radiation therapy and how it hurts to move or to be touched. Moreover, you are still expected to go to work during all of this with little to no help with bills from the programs in place for cancer patients. I was grateful for the $125 I received every two weeks, but what actual bill could I pay with that seriously?"

© 2025 Bhatt NS et al. *JAMA Network Open*.

eAppendix. Imputation

Imputation was carried out using predictive mean matching. Two rounds of imputation were conducted – the first to address missing values in demographic variables such as household income and marital status, and the second to address missing values in employment variables. Variables used for imputation in the demographic round included demographic variables, disease related variables, and select work-related variables. There were few missing values for the demographic variables (see Table 1), so 5 imputed datasets were created for regression analyses, and were pooled using Rubin's rules to obtain pooled estimates, confidence intervals, and p-values. The n for these datasets was 198. Variables used in the employment round included the same variables as above, plus a more complete list of work-related variables. Students and unemployed were excluded, as well as several participants who had all missing responses for the work-related variables. Due to the higher number of missing values in the employment related questions (see Table 2), 50 imputed datasets were created. The n for these datasets was 161. The number of datasets was verified using the "howManyImputations" R package (https://cran.r-project.org/web/packages/howManyImputations/index.html), and imputation was carried out using the "mice" package (https://cran.r-project.org/web/packages/mice/index.html). Analyses involving a work-related variable as a covariate or outcome used the 50 n=161 datasets. All other analyses used the 5 n=198 datasets. A full list of variables for each round is found below.

Variables used for imputation of work-related variables:
- Age at diagnosis
- Age at survey
- Sex
- Race
- Ethnicity
- Marital status
- Education
- Household income
- Disease type
- History of chemotherapy exposure
- History of radiation therapy exposure
- History of surgery
- History of hematopoietic cell transplant
- How many hours does your employer expect you to work in a typical 7-day week?
- About how many hours altogether did you work in the past 4 weeks (28 days)?
- On a scale from 0 to 10 where 0 is the worst job performance anyone could have at your job and 10 is the performance of a top worker, how would you rate the usual performance of most workers in a job similar to yours?
- Using the same 0-to-10 scale, how would you rate your usual job performance over the past year or two?
- Using the same 0-to-10 scale, how would you rate your overall performance on the days you worked during the past 4 weeks?
- Are you working a full-time paying job?
- Average number of hours working a full-time paying job?
- Are you working a part-time paying job?

© 2025 Bhatt NS et al. *JAMA Network Open*.

- Average number of hours working a part-time paying job?
- In the past 28 days, how many days did you:
    - Miss an entire workday because of problems with your physical or mental health?
    - Miss an entire workday for any other reason (including vacation)?
    - Miss part of a workday because of problems with your physical or mental health?
    - Miss part of a workday for any other reason (including vacation)?

Variables used for imputation of demographic variables:

- Age at diagnosis
- Age at survey
- Sex
- Race
- Ethnicity
- Marital status
- Education
- Household income
- Disease type
- History of chemotherapy exposure
- History of radiation therapy exposure
- History of surgery
- History of hematopoietic cell transplant
- Current work status
- How many hours does your employer expect you to work in a typical 7-day week?
- About how many hours altogether did you work in the past 4 weeks (28 days)?
- On a scale from 0 to 10 where 0 is the worst job performance anyone could have at your job and 10 is the performance of a top worker, how would you rate the usual performance of most workers in a job similar to yours?
- Using the same 0-to-10 scale, how would you rate your usual job performance over the past year or two?
- Using the same 0-to-10 scale, how would you rate your overall performance on the days you worked during the past 4 weeks?
- Are you working a full-time paying job?
- Average number of hours working a full-time paying job?
- Are you working a part-time paying job?
- Average number of hours working a part time paying job?

© 2025 Bhatt NS et al. *JAMA Network Open*.
