# Supplementary material for: Work Status, Absenteeism, Presenteeism, and Quality of Life in Young Adult Cancer Survivors
Source: JAMA Netw Open. 2025 Aug 26;8(8):e2528882. doi: 10.1001/jamanetworkopen.2025.28882 (PMC12381670; doi:10.1001/jamanetworkopen.2025.28882)
Supplement: Supplement 2. — Data Sharing Statement [file jamanetwopen-e2528882-s002.pdf]

# Data Sharing Statement

Bhatt. Work Status, Absenteeism, Presenteeism, and Quality of Life in Young Adult Cancer Survivors. *JAMA Netw Open*. Published August 26, 2025.
doi:10.1001/jamanetworkopen.2025.28882

**Data**
**Data available:** Yes
**Data types:** Deidentified participant data, Data dictionary
**How to access data:** Interested individuals can contact the corresponding author regarding de-identified participant data and data dictionary.
**When available:** With publication

**Supporting Documents**
**Document types:** None

**Additional Information**
**Who can access the data:** Researchers whose proposed use of the data has been approved
**Types of analyses:** Interested individuals can contact the corresponding author regarding any potential secondary analyses
**Mechanisms of data availability:** After approval of a proposal and with a signed data access agreement
